# MEK inhibition in adult patients with pilocytic astrocytomas

**DOI:** 10.1038/s41698-026-01334-z

**Published:** 2026-03-16

**Authors:** Konstantinos Rounis, Anna Falk Delgado, Christopher Illies, Alia Shamikh, Theresa Wangerid, Margret Jensdottir, Jiri Bartek, Oscar Persson, Max Albert Hietala, Signe Friesland, Hanna Carstens, Giuseppe Stragliotto

**Affiliations:** 1 Karolinska Comprehensive Cancer Center, Solna, Sweden; 2 Department of Oncology-Pathology, Karolinska Institutet, Stockholm, Sweden; 3 Department of Neuroradiology, Karolinska University Hospital, Solna, Sweden; 4 Department of Clinical Neuroscience, Karolinska Institutet, Solna, Sweden; 5 Department of Oncology-Pathology, Karolinska University Hospital, Stockholm, Sweden; 6 Department of Neurology, Karolinska University Hospital, Stockholm, Sweden; 7 Department of Neurosurgery, Karolinska University Hospital, Stockholm, Sweden

**Keywords:** Cancer, Neurology, Oncology

## Abstract

Pilocytic astrocytomas (PAs) are rare primary brain tumors in adults and exhibit worse outcomes compared with pediatric cases. Surgery is the primary therapeutic approach, whereas radiation therapy (RT) is reserved for symptomatic recurrence but is associated with substantial long-term toxicity. PAs are characterized by the activation of the mitogen-activated protein kinase (MAPK) signaling pathway and Mitogen-activated protein kinase kinase (MEK) inhibitors have demonstrated effectivity in pediatric populations, though data in adults remain limited. We retrospectively analyzed data of five adult patients (ages 30–79), four with PAs and one with PA with anaplastic transformation, that were administered trametinib, a MEK1/2 inhibitor. All tumors harbored somatic *NF-1* mutations, with one germline NF-1 case. According to RANO 2.0 criteria, three patients achieved partial responses and two had stable disease, with neurological improvement in two cases. Median progression-free survival was 16.65 months, supporting MEK inhibition as an effective treatment strategy in adult PAs.

## Introduction

Pilocytic astrocytoma (PA) is a WHO grade 1 primary brain tumor that comprises approximately 2–3% of all primary Central Nervous System (CNS) malignancies^1,2^. These uncommon tumors are characterized by a bimodal age distribution, with the vast majority of new cases being diagnosed before the age of 19 years old^3^. PAs are rare in patients older than 40 years old, with only 275 patients reported in the SEER database from 1973 to 2008^4^.

PAs in children have a favorable prognosis with a 5 year overall survival (OS) of 95%^4^. Adult patients with PAs have a worse prognosis, and their survival has an inverse correlation with age, with 5 year OS ranging from 89.5% in individuals aged 20–39 years old, 77% in those aged 40–59, and 52.9% in patients older than 60 years old^4^. Unfortunately, because of the rarity of this tumor entity in adult individuals the majority of data addressing treatment and prognosis are extrapolated from data from children.

Complete surgical excision is associated with an excellent prognosis^5^ but even individuals who underwent incomplete excision can have a favorable clinical outcome^5^. In the setting of symptomatic disease progression after incomplete excision, radiotherapy (RT) is the mainstay of treatment, with 10 years progression free survival (PFS) exceeding 70%^6,7^. However, RT is associated with devastating late side effects^8,9^, especially in children and young adults. Furthermore, it carries a notable risk of malignant transformation from a pilocytic astrocytoma to a high-grade glioma^10^. The risk for significant neurological sequalae as a result of RT administration has led clinical practice to administer systemic therapy as the preferred initial treatment of choice, in the case of symptomatic progression not amenable to surgical excision, in order to prolong RT administration in children and young adults. However, cytotoxic chemotherapy in patients with PAs has demonstrated modest clinical efficacy^11–13^. A retrospective analysis of 31 adult patients with PAs that were treated with chemotherapy performed by Berg et al.^12^ reported a median Progression Free Survival (PFS) of 20 months and a median OS of 49 months. Kesari et al.^13^ in a phase II study investigated the effect of protracted temozolomide administration in 44 adult patients with PAs. Protracted temozolomide administration was associated with a median PFS of 38 months^13^. However, due to the rarity of the disease, the limited number of patients reported, and the lack of detailed molecular data in these studies, it remains difficult to draw reliable conclusions regarding the effectiveness of cytotoxic chemotherapy in adult patients with PAs.

From a molecular standpoint, PA pathogenesis is characterized by the aberrant activation of the Mitogen-Activated Protein Kinase (MAPK) pathway. Comprehensive genomic analysis has demonstrated that single abnormalities of the genes involving the MAPK pathway are found in almost all cases of PAs^14^. The most common genetic abnormalities involve genomic aberrations in the v-Raf murine sarcoma viral oncogene homolog B (*BRAF*) and the neurofibromin-1 (*NF-1*) gene^14,15^.

Mitogen-activated protein kinase kinase (MEK) is a crucial downstream mediator of the MAPK intracellular signaling cascade^16^. Its inhibition, in MAPK activated tumors, has demonstrated interesting results in the setting of phase I trials^17^, and it does not pose the risk of paradoxical MAPK pathway activation that has been reported with the administration of single second generation BRAF inhibitors such as dabrafenib or encorafenib^18^. Selumetinib, a MEK 1-2 inhibitor, has demonstrated positive results in children with MAPK pathway activated low-grade gliomas (LGG)^17,19–22^ and with NF-1 related inoperable neurofibromas^23^. Trametinib, another MEK 1-2 inhibitor, has also demonstrated promising activity in children and young adults with PAs^24–28^. Furthermore, in children with LGG harboring the *BRAF* V600E mutation, the administration of dabrafenib in combination with trametinib was superior in terms of response rates and PFS in comparison to standard chemotherapy with carboplatine-vincristine^29^. Finally, the FIREFLY-1 phase II trial investigated the effect of tovorafenib, a type II RAF inhibitor, in patients with LGG with *BRAF* fusions or mutations^30^. Tovorafenib administration was associated with high response rates (RR) at the level of 67% and a duration of response (DOR) of 16.6 months^30^.

However, despite the encouraging results on the effectivity of MAPK pathway inhibition on PA in children, data in adults with PAs are lacking. Advanced age has been a well-recognized factor of adverse clinical outcomes in PAs^31^, but comprehensive comparative genomic analysis in adult patients with PA has not revealed any major differences in the spectrum of MAPK alterations in these individuals^3,32^. The preceding finding leads to the conclusion that the inhibition of the MAPK pathway in adult patients with PAs would potentially yield positive outcomes.

In order to further address this hypothesis, we retrospectively analyzed data of 5 adult patients (range: 30–81 years old), with pilocytic astrocytomas harboring NF-1 mutations, who received trametinib as an off-label treatment in our center.

## Results

### Patient characteristics and disease trajectory

Patients’ characteristics are depicted in Table 1. Five patients in our center received treatment with trametinib 2 mg daily initially, either as salvage treatment for heavily pretreated PAs or in order to postpone or avoid RT administration. Four out of five patients were alive at the time of data cut-off. The patients 1–4 in our cohort were male, and patient 5 was female. The median age of our patients at the time of trametinib administration was 36 years old (range 30–79 years old). Out of all the patients, only patient 3 had received a diagnosis of PA as a child at the age of eight years old. In addition, only patient 3 had a diagnosis of neurofibromatosis type 1. Two patients in our cohort had infratentorial tumors, whereas, the rest had supratentorial tumors (Table 1). All of the patients experienced neurological symptoms as a result of their CNS malignancies. Patients 1 and 2 experienced balance problems because of their tumor’s localization in the right cerebellar hemisphere. Patient 3 has serious developmental and memory problems, and patient 4 suffers from aphasia and balance problems. Patient 5 experienced visual disturbances in her left peripheral visual fields. All the patients had adequate Karnofsky Performance Score (KPS) in order to be administered systemic treatment.

**Table 1:** Patient characteristics

Patient	Age^*1^	Histology (grade)	Ki-67	Tumor location	MGMT methylation status	KPS^*3^	Previous treatments^*4^	Neurofibromatosis type 1	*NF-1* mutation (VAF^*5%^)	Additional genomic aberrations (VAF %)	Days under trametinib treatment	Best response to trametinib (RANO)	Change of contrast enhancing lesions under trametinib (RANO 2.0)	Progress under trametinib	Neurol-ogical benefit	Toxicity	PFS^*7^ (months)	Follow up (months)
1	74	PA (I)	2%	Right celebellar middle penducle	Methylated	80	Biopsy, TMZ	No	Exon 32, c4206 del, p.Ile1402MetfsTer4 (15%)	None	554	PR	−50%	Yes	Yes	Grade III rash	16.65	26.43
2	79	PA (I)	9%	Right celebellar hemisphere	Methylated	90	Biopsy	No	Intronic c.6819+3 A > G (63%)	*1) BRAF*: exon15c.1780 G > A pAsp594Asn (14%) *2) PTPN11*: exon 3 c227A>C p. Glu76Ala (5.25%)	182	PR^*6^	−74%	Yes	No	Grade III rash	5.96	21.61
3	36	PA (I)	3%	Mesencephalon	Not methylated	80	GTR, RT, TMZ, CPT-11, bevacizumab	Yes	1) Exon 50, c7267_7268insAA, p.Thr2423fs (53%) 2) Exon 13, c1467T>A, pTyr489Ter (11%)	None	277	SD	−17%	No	No	Grade III rash	15.64	19.44
4	30	Astrocytic tumor with pilocytic features or PA with malignant tranformation^*2^ (IV)	20%	Left thalamus	Methylated	70	GTR x 2, RT, TMZ, CCNU, bevacizumab	No	Exon 16 c1729_1730insA pMet577fs (22%)	*1) CDKN2A*: Exon 2 c172_176delCGAGT p.Arg58fs (7%) *2) CBL*: Exon 8 c.1111 T > C p.Tyr371His (4.3%) *3) BRAF* amplification	598	PR	-55%	No	Yes	Grade III rash	17.70	19.41
5	29	PA (I)	2%	Left uncus	Not methylated	90	GTR x 2	No	Exon 10 c1166A>G, pHis389Arg (49%)	*CIC*: Exon 20 c4534C>T, p.Arg1512Cys (4%) (Detected only after the first surgery)	265	SD	+23%	No	No	Grade III rash	10.98	17.96

^*1^: Age at the beginning of trametinib treatment ^*2^: The patients’ tumor was unclassified despite further methylation analysis. The diagnosis falls between astrocytic tumor with pilocytic features [cIMPACT-NOW, update 6 (2020)] or pilocytic astrocytoma with anaplastic transformation (WHO 2016) ^*3^: *KPS* Karnofsky Performance Score ^4*^: Before the administration of trametinib ^5*^: *VAF* Variant allele frequency ^6*^: The patient had increase of the max diameter of his tumour due to expansion of the outer borders because of necrosis of the central area but the total sum of the contrast enhancing areas was reduced, ^7*^: *PFS* Progression Free Survival.

*PA* Pilocytic astrocytoma, *TMZ* Temozolomide, *GTR* Gross tumor resection, *RT* Radiation therapy, *CPT-11* Irinotecan, *CCNU* Lomustine.

**Fig. 1:**
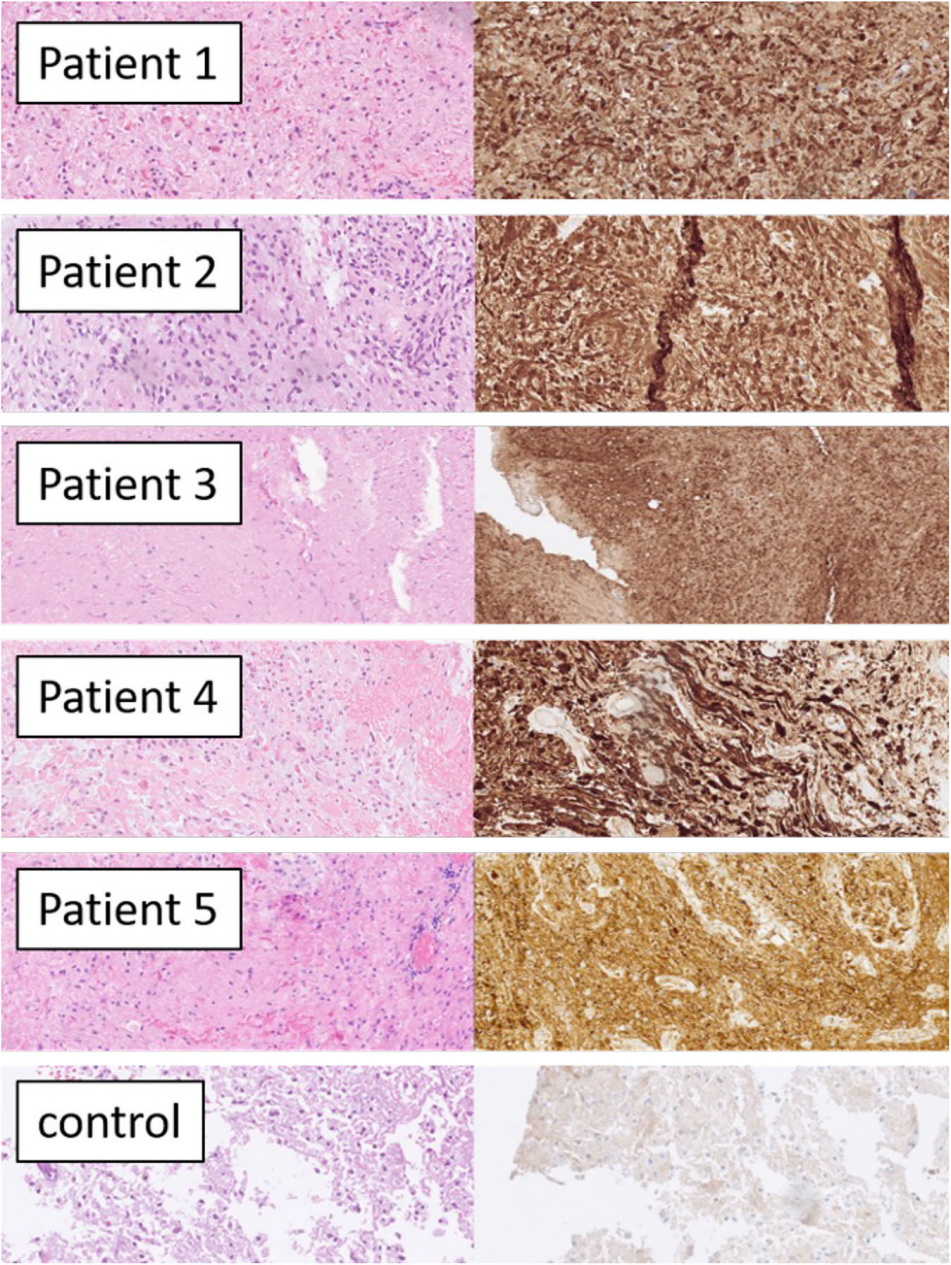
Histology of all tumors and control tissue (gliosis): H&E (left) and immunohistochemical stain for p-ERK (Thr202/Tyr204, D13.14.4E, Cell Signaling, right), all from scanned slides, all 20 x objective.

The decision for trametinib administration was taken by the multidisciplinary neuro-oncological conferences in our center. The decision was based due to existing publications demonstrating efficacy of MEK inhibitors for the treatment of PAs in children and young adults^20,21,24,26^ and the available data demonstrating similar genomic landscape between PAs in adults and PAs in children^3,32^. Furthermore, all tumors in our cohort demonstrated positive p-ERK staining (Fig. 1), and each harbored mutations associated with MAPK pathway activation. For Patients 1 and 2, gross total resection was not feasible due to tumor location. In addition, given their advanced age, radiation therapy was not pursued. Patient 1 had previously received temozolomide, achieving radiological stability but experiencing neurological deterioration after three cycles, prompting a switch to trametinib. Patient 2, who presented with a MAPK-activated cerebellar lesion and preserved performance status for his age, was started on trametinib as first-line therapy. Patients 3 and 4 were heavily pretreated, having progressed following radiotherapy and two lines of cytotoxic chemotherapy. MEK inhibition was therefore initiated as salvage therapy due to the lack of remaining treatment options. Patient 5 experienced tumor progression after two gross total resections; because of the tumor’s location near the amygdala and the absence of MGMT promoter methylation, MEK inhibition was selected with the goal of delaying the need for radiotherapy. A treatment timeline summarizing the therapeutic course for all patients is provided in Fig. S1.

### Histology and molecular analysis

All of the patients’ tumors were classified as PAs with the exception of patient 4. Diagnosis was based on histology according to the criteria of WHO valid at the time of diagnosis. Except for case 2, all tumors had Rosenthal fibers and/or eosinophilic granular bodies (EGBs). The tumor of patient 2 was dominated by an oligodendrocyte-like morphology. Except from case 4, all the patients’ tumors were classified as PAs after the first surgery/biopsy, and the diagnosis was confirmed for patients 3 and 5 after the second surgery. More specifically, the patient’s 4 tumor was classified after the first surgery as a heterogeneous tumor with cystic formation, phospho-ERK(p-ERK) positive, with a Ki-67 lower than 2%. Molecular analysis in the surgical material after the first operation demonstrated only a mutation in the *ATRX* gene (Exon 17c.4741_4745delAAAC p.Thr.1582 fs). The tumor of patient 4 was classified as PA with anaplastic transformation (WHO 2016)^33^ or as an astrocytic tumor with piloid features [(cIMPACT-NOW, update 6 (2020)]^34^ after the second operation. The ki-67% after the second operation was increased at the level of 20%. The molecular analysis that was performed after the second operation did not demonstrate the previously found mutation in the *ATRX* gene, but new mutations in the *NF-1*, *CDN2A,* and *CBL* genes and *BRAF* gene amplification (Table 1). Methylation analysis was conducted for the further classification of the patient’s tumor (Illumina EPIC 850k Beadchip), analysis with Heidelberg classifier v11b4 was inconclusive, with no score higher than 0.5. Highest match (score 0.47) was to “methylation class family pilocytic astrocytoma”. The CNV plot showed a gain in the BRAF region of chromosome 7q and no convincing losses.

Somatic *NF-1* mutations were identified through NGS application in the PAs of all the patients in our cohort. Patients 2 and 4 had a more complex genomic landscape. More specifically, patient’s 2 tumor had an intronic *NF-1* mutation with a variant allele frequency (VAF) of 63%, a class III *BRAF* mutation in exon 15 of the *BRAF* gene (D594N) (VAF = 14%), and a subclonal mutation in the third exon of the *PTPN11* gene (VAF = 5.25%). Patient 4 had additionally to the mutation in *NF-1* gene, mutations in the *CDKN2A* and *CBL* genes and gene amplification of the *BRAF* gene. The patient’s 5 tumor had a mutation of *NF-1* gene with high VAF at the level of 49%, with unknown clinical significance, and a mutation in the *CIC* gene with low VAF (4%) was detected after the first surgery of the patient, but it was not detected in the surgical material after the second surgery. Only patient 3 had a clinical diagnosis of neurofibromatosis type 1. None of the other patients in our cohort had any clinical signs of an underlying hereditary NF-1 mutation or positive family history, and this is the reason why further germline testing or investigation for potential mosaicism was not conducted.

### Assessment of MEK inhibition efficacy

**Fig. 2:**
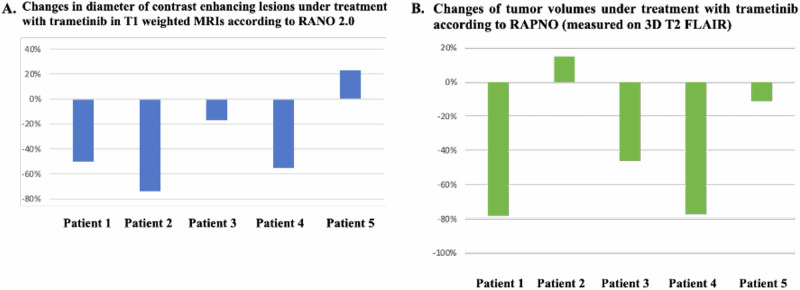
Radiological assesment of MEK inhibition effi cacy in our patient cohort. **A** Percentage of maximum tumor diameter (contrast enhancing lesions) change under treatment with trametinib in our patient cohort. **B** Changes of tumor volumes under treatment with trametinib as measured on 3D T2 FLAIR.

Three out of five patients in our cohort experienced partial responses (PR) according to RANO 2.0 and two had stable disease as best response under treatment with MEK inhibition (Fig. 2A). Evaluation of tumor volumes under trametinib measured on 3D T2 FLAIR demonstrated that all patients except patient 2 experienced tumor shrinkage (Fig. 2B). More specifically, patient 1 and 4 experienced PR with tumor volume reduction 78% and 77% respectively as best response to treatment (Table S1). Patient 3 had a minor response (MR) according to RAPNO criteria^35^ with a tumor volume reduction of 46% (Table S1). Patient 5 had a tumor volume reduction of 11% that is classified as stable disease (SD), and patient 2 experienced an increase in tumor volume at the level of 15% as the best response to treatment, which is also classified as SD^35^ (Table S1). Trametinib administration was associated with prolonged disease control with a median PFS of 16.65 months (95% CI: 7.81–25.49) in our cohort.

**Fig. 3:**
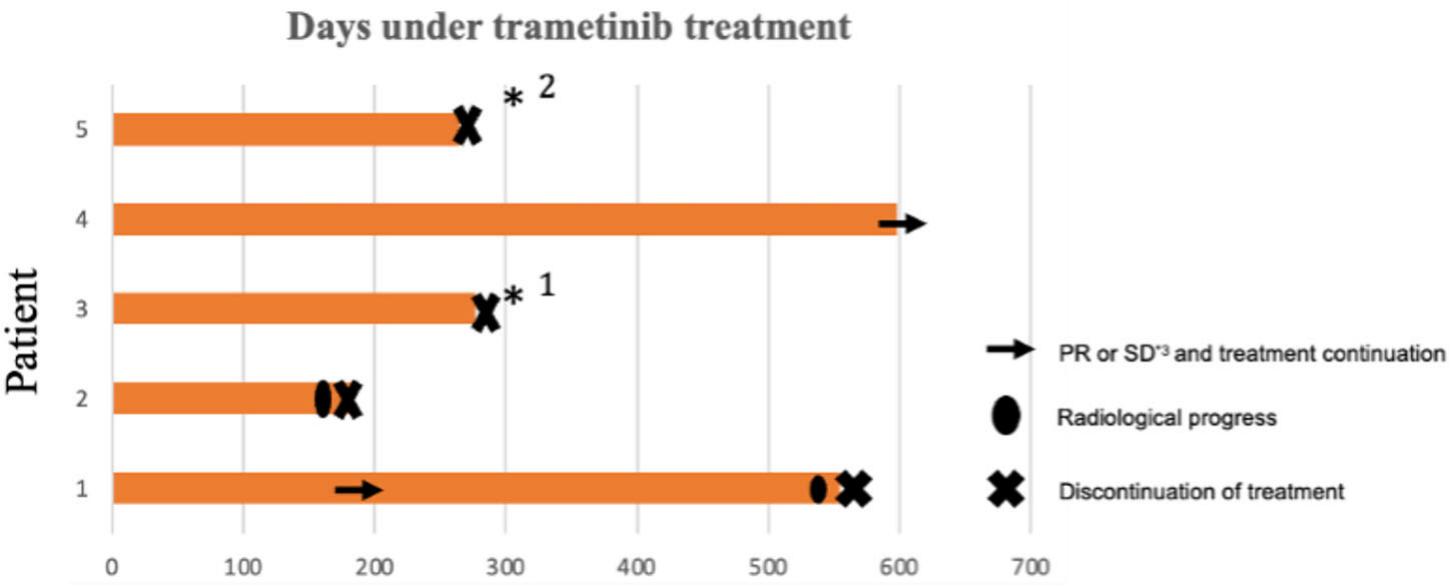
Time receiving MEK inhibition (trametinib) for the five patients in our cohort. Only 1 out of 5 patients was under treatment at the time of data cut-off. *1: Patient 3 discontinued trametinib due to toxicity, *2: Patient 5 discontinued trametinib due to pregnancy.

Patients 1 and 2 discontinued trametinib because of progression (Fig. 3). Patient 3 discontinued treatment because of grade III rash and grade III peripheral edema despite dose reductions and concomitant treatment with iso-tretinoin. Patient 5 voluntarily ceased trametinib therapy in view of her intention to achieve pregnancy. Patient 4 is still under treatment (Fig. 3).

**Fig. 4:**
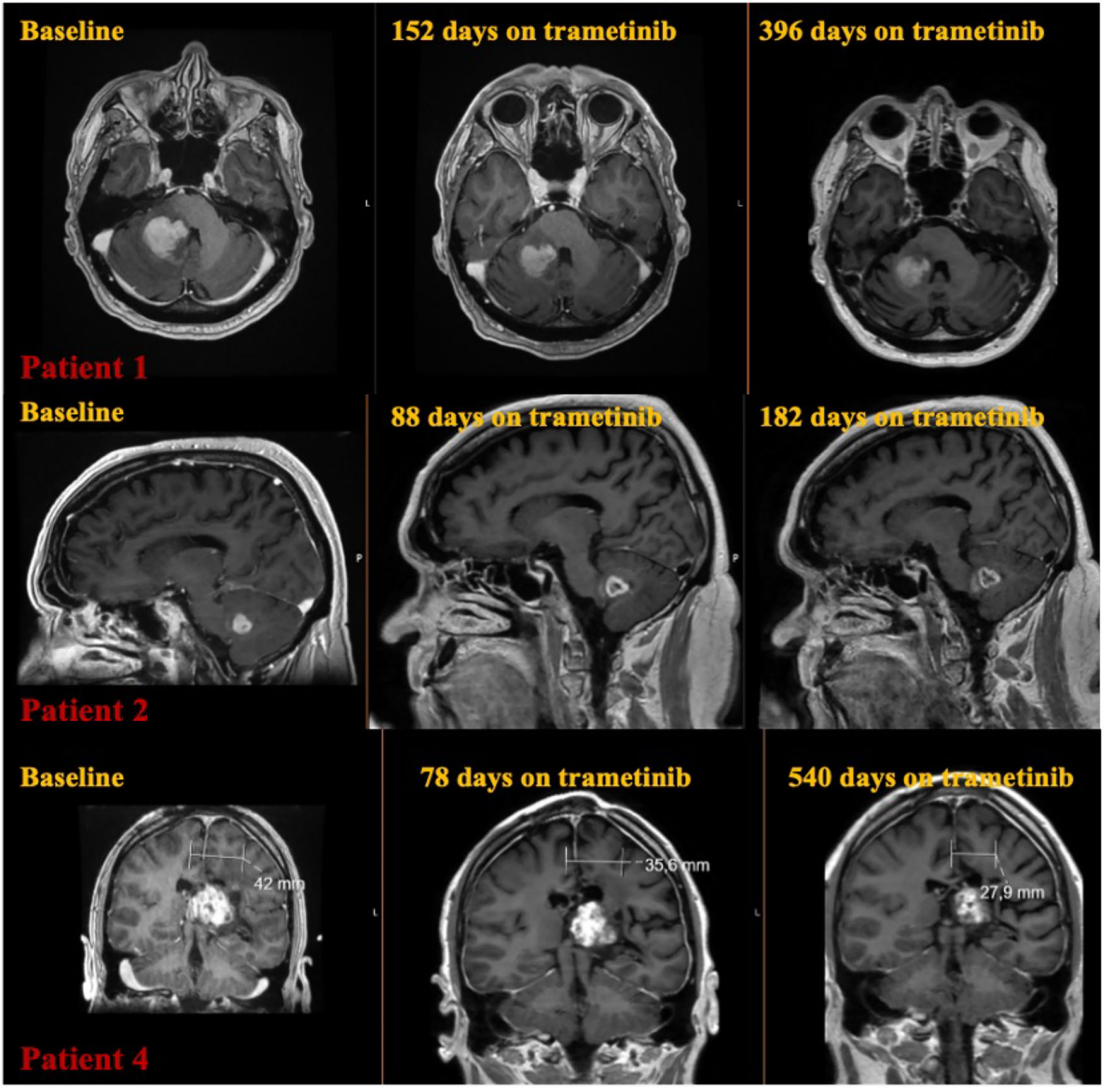
T1 weighted MRI images with contrast enhancement demonstrating the fluctuation of tumor growth in patients 1, 2, and 4 of our cohort under trametinib treatment. Baseline MRI means MRI control before the start of MEK inhibitor.

In patient 2, the outer borders of the tumor had been expanded during the first control because of necrosis of the central area, but the sum of all the contrast enhancing lesions had been reduced (Figs. 2 and 4). The patient at the time was neurologically stable, and it was decided from the neurooncological multidisciplinary conference to continue with trametinib. Trametinib was discontinued after the patient had neurological deterioration at the time of the second control. Radiologically, he had further progression of the central necrosis and progress of the non-enhancing areas of the tumor, and treatment was subsequently switched to temozolomide.

Two out of the five patients in our cohort experienced significant neurologic improvement under treatment with MEK inhibitor. Patient 1 experienced a significant improvement with his movement difficulties, and he was able, after 3 months of treatment, to become independent from the use of a wheelchair (Fig. 4). Patient 4 had significant problems of language comprehension. After four months of treatment, he experienced noticeable improvement of his language comprehension capabilities (Fig. 4). Patients 3 and 5 had stable neurological symptoms under treatment and patient 2 deteriorated neurologically after approximately 5 months of trametinib treatment.

### Toxicity

All the patients in our cohort started treatment with trametinib at 2 mg daily. They all experienced grade III skin toxicity with maculopapular rash, which was managed successfully with temporary treatment discontinuation, antibiotics, and per os prednisolone treatment. Trametinib treatment was reinstituted to all the patients at a lower dose of 1 mg daily. Patient 3 demonstrated grade III rash even at the dose of 1 mg, and in order to continue his treatment iso-tretinoic acid was administered concomitantly with trametinib. Despite the rash improvement he developed, thereafter, grade III peripheral edema, and treatment was discontinued. Although he is off treatment for several months, he has not experienced any radiological progression or worsening of his neurological status.

## Discussion

The results of this retrospective case series demonstrate that MEK inhibition therapy can be effective in adult patients with PAs. All of the patients in our cohort harbored somatic *NF-1* mutations in their tumors. Patient 3 had a clinical NF-1 diagnosis. None of the other patients has been tested for germline mutations or mosaicism due to absence of any symptoms or signs of the presence of a germline *NF-1* mutation. None of the treated patients in this cohort had *BRAF* fusions or *BRAF* V600E mutations.

All the patients’ tumors had been classified as PAs with the exception of patient 4, for which even the methylation analysis was inconclusive. Lucas et al.^36^ demonstrated through comprehensive molecular analysis of gliomas in patients with Neurofibromatosis type 1 that a second hit is required in the second *NF-1* allele for the pathogenesis of gliomas in this subset of individuals^36^. In our cohort this is consistent with the genomic findings of patient 3, with a germline mutation in exon 50 of *NF-1* and a second hit with a somatic mutation in exon 11 in the other allele. However, in the rest of the patients, the molecular pathogenesis is not as clear. Molecular analysis of the tumors for patients 1 and 5 demonstrated only somatic mutations in a single allele in *NF-1* without any additional genomic aberrations in the MAPK pathway. Furthermore, patient 2 had an *NF-1* intronic mutation with high VAF at 63% and *BRAF* type III mutation at low VAF at 14%. *BRAF* type III mutation could potentially act as a single oncogenic driver for this patient, but the low VAF in comparison with the NF-1 mutation might indicate that this mutation might be subclonal, thus, it does not provide a robust explanation. Finally, patient 4 had an *NF-1* mutation with VAF of 22% and two subclonal mutations of *CDKN2A* and *CBL* plus *BRAF* amplification. The CNV plot demonstrated a gain in the BRAF region of chromosome 7p without any additional losses. Despite the fact that an additional molecular event was not found in the patients’ (except in patient 3) tumors to provide a clear molecular pathogenetic mechanism, all the patients tumors stained positive for p-ERK (Fig. 1), finding consistent with MAPK pathway activation. These results can be explained as the analysis with the NGS panel used does not provide a full exon coverage of the genes involved in the MAPK pathway, as it does not detect additional genomic aberrations such as specific epigenetic modifications (table S2). Also, absence of other mutations (t ex, IDH1/2, histone 3, TP53, or TERT-promoter) served to exclude other entities.

In our cohort, MEK inhibition led to three out of five patients to achieve PR as best response to treatment, and the rest two to have SD as best response according to RANO criteria^37^. Interestingly, further analysis of the tumor volumes in T2 3D FLAIR demonstrated tumor shrinkage in four out of five patients. Patient 2 had PR according to RANO^37^, but his tumor volume had increased in size in the T2 3D FLAIR measurement. The increase of the tumor volume in T2 3D FLAIR was reflected clinically since he was the only patient in our cohort who experienced neurological deterioration under MEK inhibition in less than 6 months. It has been described previously that MEK inhibition can lead to a decrease in contrast enhancement of the tumors without any decrease in size^35,38^. These findings indicate that additional radiological analyses based on tumor volume might be necessary for the proper evaluation of efficacy of targeted treatments in these patients.

MEK inhibition demonstrated approximately the same effectivity in adult patients with PAs in our cohort when compared to previously published reports in children and young adults^19,20,24,26,28^ (Table 2), however, due to the low number of patients, our results should be interpreted with cautiousness. Nevertheless, the patients in our cohort experienced a PFS higher than 12 months (16.65 months), and 4 out of 5 patients had a tumor volume reduction in 3D T2 FLAIR. Previously published reports in patients younger than 18 years old have shown a median PFS higher than 12 months^24,26,30^ and Baherjee et al.^19^ and Fangusaro et al.^20^ demonstrated a median PFS higher than 2 years. Furthermore, across studies in children and young adults, high rates of disease stabilization (MR or PR or SD) were achieved through MEK or BRAF type II inhibitors administration with PD as best response to treatment, ranging from 27%^24^ to 0%^26^. Our cohort provided similar results, with all the patients in our cohort achieving tumor shrinkage, with the exception of patient 2, who had an increase in his tumor volume at the level of 15% that was still classified as SD according to RAPNO^35^.

**Table 2:** Summary of clinical studies and published case series on the efficacy of MEK or RAF II inhibitor in patients with MAPK activated pediatric low grade gliomas

Study	Diagnosis	Number of patients	Agent	Median age^*8^	Genomic lesions	Response rates^*9^	Median PFS^*10^
Banerjee et al.^19^	Pediatric LGG^1^ (57% of pts^2^ had PA^*3^)	38	selumetinib	13.3 years	10 pts KIAA-1549 fusion 2 pts BRAF V600E 1 pt both BRAF fusion and BRAF mutation Rest unknown genomic status	PR: 27/38 pts SD: 5/38 pts PD: 6/38 pts	> 2 years
Fangusaro et al.^20,21^	Pediatric LGG 2 cohorts: Stratum 1: PA with BRAF fusion or BRAF V600E Stratum 3: NF-1^*4^ related LGG, 4/25 pts had PA and 13/25 pts had OPG^*5^	50 (25 pts in stratum 1 and 25 pts in stratum 3)	selumetinib	Stratum 1: 9.2 years Stratum 3:10.2 years	Stratum 1: BRAF fusions or BRAF V600E mutations Stratum 3: NF-1 related (clinical or genetic) LGG	Stratum 1: PR: 9/25 pts SD: 9/25 pts PD: 7/25 pts Stratum 3: PR: 9/25 pts SD: 15/25 PD: 1/25	Stratum 1: 2 years PFS 70% Stratum 3: 2 years PFS 96%
Manoharan et al.^26^	Pediatric LGG: 6/11 pts PA 2/11 pts NOS^*6^ 3/11 pts LGG	11	Trametinib	14.7 years old (2 adult patients, 1 19 years old and 1 25 years old)	2/11 pts NF-1 (molecular) 2/11 pts NF-1 (clinical) 4/11 pts KIA1549-BRAF 1/11 pts FGFR mutation 1/11 pts heterozygous CKN2A 1/11 pts unknown	2/11 MR 2/11 PR 7/11 SD	Not reported, but 9/10 patients received trametinib for more than 12 months
Hanzlik et al.^24^	LGG: 5/11 pts PA 1/11 pts DIG^*7^ 2/11 pts Glioneuronal	11	Trametinib	8/11 patients < 18 years old 3/11 patient > 18 years old	3/11 pts KIA1549-BRAF 1/11 pts BRAF V600E 1/11 pts NF-1 (somatic) 1/11 pts NF-1 (germline)	3/11 MR 2/11 PR 3/11 SD 3/11 PD	95% CI: 10–36 months
Selt et al.^28^	Pediatric LGG: 10/18 sporadic LGG 8/18 NF-1 related LGG	18	Trametinib	8.2 years old	8/18 pts KIA1549-BRAF 3/18 pts NF-1 1/18 pts FGFR1 K654Q 1/18 pts BRAF V600E	2/11 MR 6/18 PR 10/18 SD	Not reported
Kilburn et al.^25^	Pediatric LGG	137	Tovorafenib (RAF II inhibitor)	9 years old	74% of pts KIA1549-BRAF 16% of pts BRAF V600E 10% of pts other BRAF lesion	26% MR 26% PR 30% SD 14% PD	Higher than 12 months among molecular subgroups

^*1^ *LGG* Low grade glioma, ^*2^ *pts* patients, ^*3^ *PA* pilocytic astrocytoma, ^*4^ *NF-1* Neurofibromin 1, ^*5^ *OPG* Optic pathway glioma, ^*6^ *NOS* Not otherwise specified, ^*7^*DIG* Desmoplastic infantile ganglioglioma, ^*8^ At the beginning of treatment with MAPK pathway inhibitor, ^*9^ *MR* Minor response, *PR* Partial response, *SD* Stable disease, *PD* Progressive disease, ^*10^ *PFS* Progression Free Survival.

Older age has been recognized as an independent prognostic factor for PAs^31^. PAs in children and PAs in adults have both been molecularly characterized from MAPK pathway alterations^3,32^. Despite the fact that no significant differences have been discovered between the genomic landscape of PAs in adults compared with affected children^3,32^, the effectiveness of MEK inhibition in adult patients has remained unclear. Hanzlik et al.^24^ included in their retrospective analysis three patients older than 18 years old that received trametinib. In the published report from Manoharan et al.^26^ data from two adult patients, aged 19 and 25 years old respectively, had been analyzed, and the first patient experienced a sustained minor response.

However, there has been a paucity of data on the effectiveness of MEK inhibition in adult patients with PA, especially for those who were diagnosed in adulthood and they are older than 60 years old. Our report includes two individuals older than 70 years old who were diagnosed with cerebellar PAs with *NF-1* somatic mutations. Patient 1 experienced a sustained minor response with significant neurologic benefit under MEK inhibition. Patient 2 experienced disease progression in the second radiological evaluation, but he had a more complex genetic profile with additional genomic aberrations in *BRAF* (*BRAF* D594N) and *PTPN11* genes, and treatment was discontinued after 5 months. In the clinical study of selumetinib by Fangusaro et al.^21^ the presence of *BRAF* V600E mutation was associated with shorter PFS, but these results cannot be extrapolated in the adult setting and in only one patient case with a tumor harboring a type III *BRAF* mutation.

In addition, high grade astrocytoma with piloid features^39^ or PA with anaplastic transformation are tumor entities with potential underlying genomic aberrations that can be associated with MAPK activation. Unfortunately, there have not been published any case reports or case series on the effectivity of MAPK pathway inhibition in these individuals. Bender et al.^39^ have published a case series of 6 patients with high grade astrocytoma with piloid features. One of the patients in the cohort from Bender et al.^39^ was treated with binimetinib, a MEK 1-2 inhibitor, and he experienced stable disease for seven months as best response to treatment. In our cohort, patient 4, was diagnosed with an aggressive tumor, which was difficult to classify, even after methylation analysis. The patients’ tumor had a genomic landscape with *NF-1*, *CKN2A,* and *CBL* mutations plus *BRAF* gene amplification. He was heavily pretreated with 2 operations, proton-radiation therapy, and 2 lines of previous chemotherapy. The patient has experienced a sustained response and clinical improvement under trametinib therapy. More data are needed on the establishment of MAPK pathway inhibition as a potential treatment option in patient with high grade astrocytoma with piloid features or in PAs with anaplastic transformation. However, MEK or BRAF-MEK inhibition (in the setting of BRAF V600E mutation) can be an off-label treatment option after progression to chemoradiotherapy in selected patients with the aforementioned malignant entities if they harbor MAPK pathway genomic alterations.

Moreover, patient 5 in our cohort received trametinib as an off-label treatment after tumor progression after 2 operations. She experienced symptoms of diminished peripheral visual fields in her left eye. Trametinib was administered in order to postpone radiation therapy. The patient had a prolonged disease control under trametinib therapy with good tolerance. The patient voluntarily chose to discontinue treatment with trametinib due to a desire to become pregnant. Radiation therapy has been associated with serious late adverse effects and a substantial risk of malignant transformation^40^. Consequently, delaying radiation therapy with systemic treatment as first line option has been the mainstay of treatment among clinicians in children and young adults, and further data accumulation on the effect of MEK inhibition is a positive step towards the achievement of this particular goal.

Furthermore, three out of five patients in our cohort had received previously cytotoxic chemotherapy, and patients 3 and 4 had been treated previously with radiation. Patient 1 and 3, and 4 experienced clinical and/or radiological progress when administered TMZ (Fig. S1). Patient 2 experienced tumor disease control with temozolomide, but with a short PFS less than 12 months (Fig. S1). Patients 1, 2, and 4 had MGMT promoter methylated tumors. Kesari et al.^13^ in a phase II study of protracted TMZ administration in adult patients with PAs reported the presence of MGMT methylation status as a positive prognostic factor, but the number of patients was small (20 patients) to draw any safe conclusions for the utility of the MGMT promoter methylation as a biomarker of chemotherapy efficacy for this subset of patients.

Finally, all the patients in our cohort experienced maculopapular rash grade III under trametinib 2 mg. The skin toxicity was much higher than that reported in the studies with children, but it can be attributed to the older age of the participants, compared with the children population. However, it was successfully managed with the administration of local treatment options, per os antibiotics, and steroids, and it was reinstituted to four out of five patients in our cohort at a lower dose of 1 mg with good tolerance.

Our data reinforce previous clinical reports^17,20–22,26–30,41^ on the effectivity of MAPK pathway inhibition as a salvage treatment or as a means to prolong RT in children and young adults. The choice between administration of cytotoxic therapy or a molecular targeted agent a first systemic treatment option is based on experts’ opinion, treating center preferences, and individual patient decisions^42^. This dilemma can be more relevant for children since data in adult patients with PAs on the effectivity of cytotoxic chemotherapy with carboplatin/vinblastine are lacking, and temozolomide with or without bevacizumab has demonstrated modest results^11–13^. An ongoing randomized trial is currently investigating the efficacy of selumetinib in comparison with the carboplatin/vinblastine combination for non-*BRAF* V600E mutant, non-NF-1 related newly diagnosed or previously untreated LGG (NCT04166409). Furthermore, new novel agents that act as inhibitors of the MAPK pathway are under investigation. Mirdametinib is a MEK ½ inhibitor that has recently gained approval for the treatment of NF-1 related neurofibromas^43^ and has also demonstrated favorable brain penetrance, a characteristic that can be relevant for future clinical trials in the therapeutic setting of MAPK activated gliomas^44^. ERK inhibitors are also an interesting class of drugs as they have demonstrated in preclinical models of pediatric low-grade gliomas harboring BRAF V600E mutations or BRAF-KIA 1549 fusions to inhibit the MAPK pathway without paradoxical activation^45^. Clinical trials of ulixertinib, an ERK inhibitor for patients with tumors harboring activating alterations of the MAPK pathway, are currently ongoing (NCT04488003, NCT03698994).

This report provides important insights into the therapeutic efficacy of MEK inhibition in adult patients with PA, addressing a clinical area in which published data are very limited. Prospective data and future clinical studies are needed to further establish the role of MEK inhibition as a treatment option within the disease trajectory, but given to the rarity of the disease, it would require a multicenter collaboration.

The limitations of the study include the small number of patients, its retrospective nature, the heterogenicity of the studied population, and the absence of patients with PAs harboring *BRAF* gene alterations. In addition, a serious limitation consists of the absence of germline genomic testing in the patients of our cohort.

## Methods

### Ethical approval and patient consent

This study was conducted in accordance with the Declaration of Helsinki and was approved by the Swedish Ethical Review Authority (approval ID: Dnr 2025-01466-01).

Written informed consent was obtained from all participants for the retrospective analysis of their clinical data and for the publication of related clinical information. For participants who were deceased at the time of data collection, consent for publication was obtained from the individual’s next of kin.

### Case series

From December 2022 until January 2024, five adult patients with PAs received treatment with trametinib in Karolinska Comprehensive Cancer Center. The decision for the administration of trametinib was decided from the hospital’s neuro-oncological multidisciplinary conferences.

Information about the patients’ symptom assessment and neurological outcomes was retrieved through the hospital’s electronic journals. Demographic, clinical, histological, and molecular data were also retrospectively collected.

### Radiological assessment

The patients’ radiological response to treatment was evaluated according to RANO 2.0 criteria and the tumor’s change in percent of the product of two maximal diameters under trametinib treatment. An additional radiological analysis was performed through the longitudinal evaluation of tumor volumes in 3D T2 FLAIR according to RAPNO criteria^35^ under treatment with trametinib.

### Genomic analysis

Molecular analysis was performed on the patients’ tumor samples. No germline testing was conducted. The genomic analysis in the patients’ tumors was performed through the application of NGS-Oncomine Childhood Cancer Assay (Amplicon Sequencing, Oncomine Childhood Cancer Research Assay, Ion Torrent S5, Ion Chef; Thermo Scientific). MGMT promoter methylation was assessed with the MGMT Pyro Kit (QIAGEN).

## Supplementary information

Supplementary information

## Data Availability

All data are presented in the current paper and the supplementary information.
