# Supplementary material for: MEK inhibition in adult patients with pilocytic astrocytomas
Source: NPJ Precis Oncol. 2026 Mar 16;10:134. doi: 10.1038/s41698-026-01334-z (PMC13022286; doi:10.1038/s41698-026-01334-z)
Supplement: Supplementary file 1 — Supplementary information [file 41698_2026_1334_MOESM1_ESM.pdf]

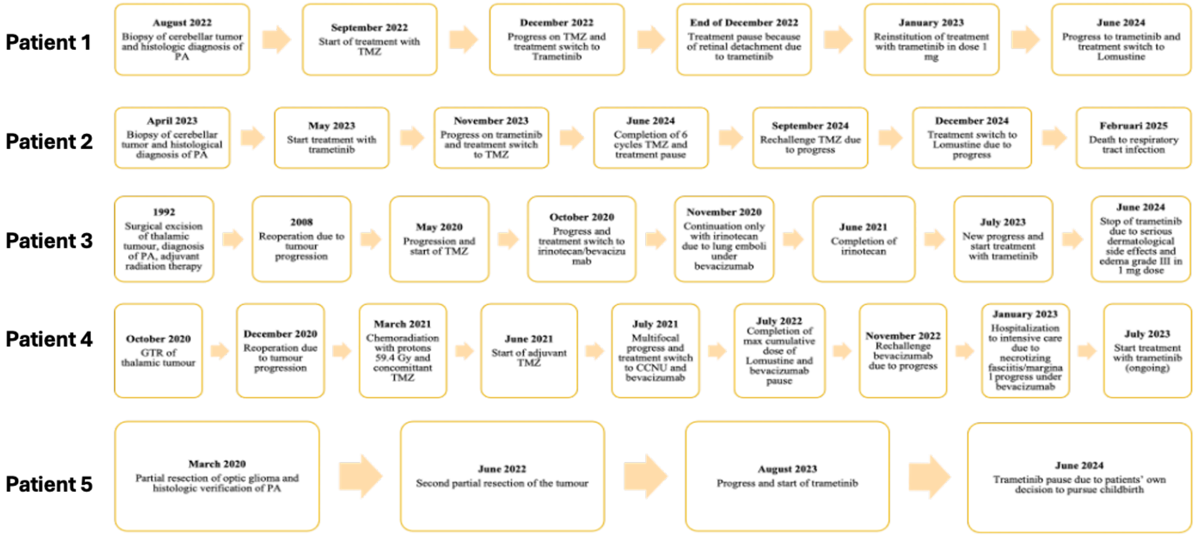

Figure S1: Horizontal timeline chart of the disease trajectory and the administered treatments of the patients in our cohort.

Patient ID	Start trametinib	Stop trametinib	Baseline scan before trametinib initiation	Last scan on MEK inhibition until 2025-04-11	Baseline scan measure (AP, trans, CC)	Follow up 1	Follow up 2	Follow up 3	Follow up 4	Follow up 5	Best radiological response	Date of best response
1	Jan-23	Jul-24	2022-11-20	2024-05-06	34x36x32=39168	25x28x28=19600	29x27x28=21924	20x23x25=11500	24x17x21=8568	NA	PR (minus 78% T2FLAIR3D)	2024-05-06
2	May-23	Dec-23	2023-03-30	2023-11-10	22x15x20=6600	21x19x19=7581	NA	NA	NA	NA	SD (plus 15% T2FLAIR3D)	2023-08-09
3	Jul-23	May-24	2023-06-14	2024-04-02	20x18x15=5400	17x13x19=4199	16x12x19=3648	16x13x14=2912	NA	NA	MR (minus 46% T2FLAIR3D)	2024-04-02
4	Aug-23	Ongoing	2023-07-04	2025-01-20	37x36x34=45288	29x27x30=23490	26x25x25=16250	25x23x26=14950	23x20x25=11500	23x20x25=11500	PR (minus 77% T2FLAIR3D)	2025-01-20
5	Sep-23	Jun-24	2023-09-15	2024-08-12	20x13x15=3900	13x19x15=3705	13x19x15=3705	13x19x14=3458	20x15x17=5100	NA	SD (minus 11% T2FLAIR3D)	2024-06-03

Table S1: Depictions of tumor volumes (measurements in 3D T2 FLAIR) in the patients of our cohort during their treatment with trametinib.

Comprehensive mutation coverage (86)	CNV (28)	Full exon coverage (44)	Fusion and expression (97)	Gene expression
ABL1 CSF1R GATA2 MAP2K2 RAF1 ABL2 CSF3R GNAQ MET RET ALK CTNNB1 H3F3A MPL RHOA ACVR1 DAXX HDAC9 MSH6 SETBP1 AKT1 DNMT3A HIST1H3B MTOR SETD2 ASXL1 EGFR HRAS NCOR2 SH2B3 ASXL2 EP300 IDH1 NOTCH1 SH2D1A BRAF ERBB2 IDH2 NPM1 SMO CALR ERBB3 IL7R NRAS STAT3 CBL ERBB4 JAK1 NT5C2 STAT5B CCND1 ESR1 JAK2 PAX5 TERT CCND3 EZH2 JAK3 PDGFRA TPMT CCR5 FASLG KDM4C PDGFRB USP7 CDK4 FBXW7 KDR PIK3CA ZMYM3 CIC FGFR2 KIT PIK3R1 CREBBP FGFR3 KRAS PPM1D CRLF2 FLT3 MAP2K1 PTPN11	*ALK IGF1R BRAF JAK1 CCND1 JAK2 CDK4 JAK3 CDK6 KIT EGFR KRAS ERBB2 MDM2 ERBB3 MDM4 FGFR1 MET FGFR2 MYC FGFR3 MYCN FGFR4 PDGFRA GLI1 PIK3CA GLI2*	*APC GATA3 RUNX1 ARID1A GNA13 SMARCA4 ARID1B ID3 SMARCB1 ATRX IKZF1 SOCS2 CDKN2A KDM6A SUFU CDKN2B KMT2D SUZ12 CEBPA MYOD1 TCF3 CHD7 NF1 TET2 CRLF1 NF2 TP53 DDX3X PHF6 TSC1 DICER1 PRPS1 TSC2 EBF1 PSMB5 WHSC1 EED PTCH1 WT1 FAS PTEN XIAP GATA1 RB1*	*ABL1 FGFR2 MEF2B NUP214 SSBP2 ABL2 FGFR2 MET NUP98 STAG2 AFF3 FGFR3 MKL1 NUTM1 STAT6 ALK FLT3 MLLT10 NUTM2B TAL1 BCL11B FOSB MN1 PAX3 TCF3 BCOR FUS MYB PAX5 TFE3 BCR GLI1 MYBL1 PAX7 TP63 BRAF GLIS2 MYH11 PDGFB TSLP CAMTA1 HMGA2 MYH9 PDGFRA TSPAN4 CCND1 JAK2 NCOA2 PDGFRB UBTF CIC KAT6A NCOR1 PLAG1 USP6 CREBBP KMT2A NOTCH1 RAF1 WHSC1 CRLF2. KMT2B NOTCH2 RANBP17 YAP1 CSF1R KMT2C NOTCH4 RECK ZMYND11 DUSP22 KMT2D NPM1 RELA ZNF384 EGFR LMO2 NR4A3 RET ETV6 MAML2 NTRK1 ROS1 EWSR1 MAN2B1 NTRK2 RUNX1 FGFR1 MECOM NTRK3 SS18*	*BCL2 BCL6 FGFR1 FGFR4 IGF1R MET MYCN MYC TOP2A*

Table S2: List of the genomic alterations analyzed by the NGS panel used in the patients of our cohort (Amplicon Sequencing, Oncomine Childhood Cancer Research Assay, Ion Torrent S5, Ion Chef; Thermo Scientific)

CARE case report guidelines

CARE Checklist (2013) of information to include when writing a case report

(cc) BY-NC-ND

Topic	Item	Checklist item description	Reported on Page
**Title**	**1**	The words "case report" should be in the title along with the area of focus	7
**Key Words**	**2**	2 to 5 key words that identify areas covered in this case report	1
**Abstract**	**3a**	Introduction—What is unique about this case? What does it add to the medical literature?	2, 3
	**3b**	The main symptoms of the patient and the important clinical findings	3, 4, 5
	**3c**	The main diagnoses, therapeutics interventions, and outcomes	3, 4, 5
	**3d**	Conclusion—What are the main "take-away" lessons from this case?	7
**Introduction**	**4**	One or two paragraphs summarizing why this case is unique with references	2, 7
**Patient Information**	**5a**	Demographic information and other patient specific information	2, 3
	**5b**	Main concerns and symptoms of the patient	3, 4, 5
	**5c**	Medical, family, and psychosocial history including relevant genetic information (also see timeline).	3, 4, 5, figure S1
	**5d**	Relevant past interventions and their outcomes	3, figure S1
**Clinical Findings**	**6**	Describe the relevant physical examination (PE) and other significant clinical findings	3, 4, 5
**Timeline**	**7**	Important information from the patient's history organized as a timeline	3, figure S1
**Diagnostic Assessment**	**8a**	Diagnostic methods (such as PE, laboratory testing, imaging, surveys)	8
	**8b**	Diagnostic challenges (such as access, financial, or cultural)	Not applicable
	**8c**	Diagnostic reasoning including other diagnoses considered	3, 4
	**8d**	Prognostic characteristics (such as staging in oncology) where applicable	3, 4, 5, 6
**Therapeutic Intervention**	**9a**	Types of intervention (such as pharmacologic, surgical, preventive, self-care)	3, 4, 5
	**9b**	Administration of intervention (such as dosage, strength, duration)	3, 4, 5
	**9c**	Changes in intervention (with rationale)	3
**Follow-up and Outcomes**	**10a**	Clinician and patient-assessed outcomes (when appropriate)	4, 5
	**10b**	Important follow-up diagnostic and other test results	4
	**10c**	Intervention adherence and tolerability (How was this assessed?)	5
	**10d**	Adverse and unanticipated events	5
**Discussion**	**11a**	Discussion of the strengths and limitations in your approach to this case	7
	**11b**	Discussion of the relevant medical literature	5, 6, 7
	**11c**	The rationale for conclusions (including assessment of possible causes)	7
	**11d**	The primary "take-away" lessons of this case report	7
**Patient Perspective**	**12**	When appropriate the patient should share their perspective on the treatments they received	N

**Informed Consent**	**13**	Did the patient give informed consent? Please provide if requested . . . .**Yes**
